# Nanoparticles as an Alternative Strategy for the Rapid Detection of *Mycobacterium tuberculosis* Complex (MTBC): A Systematic Literature Review of In Vitro Studies

**DOI:** 10.1049/nbt2/4639233

**Published:** 2025-05-29

**Authors:** Abayeneh Girma, Fentaye Kassawmar, Yeshiwas Kassa, Yeshwas Asrat

**Affiliations:** ^1^Department of Biology, College of Natural and Computational Science, Mekdela Amba University P.O. Box 32, Tulu Awuliya, Ethiopia; ^2^Department of Biology, College of Science, Bahir Dar University P.O. Box 79, Bahir Dar, Ethiopia; ^3^Department of Biology, College of Natural and Computational Science, Debre Tabor University P.O. Box 272, Debre Tabor, Ethiopia

**Keywords:** disease biomarkers, immunodiagnostics, *Mycobacterium tuberculosis*, nanodiagnostics, nanoparticles, systematic review, tuberculosis

## Abstract

Worldwide, tuberculosis (TB) ranks as a second leading cause of death. The End TB strategy targets eliminating TB by 2030. Achieving this goal requires an early, accurate, and affordable diagnosis applicable in low- and middle-income countries; increasing the reach of point-of-care (POC) diagnostics is essential. Nanodiagnostics aims to enhance clinical diagnostic procedures with heightened sensitivity and accuracy by focusing on distinctive markers for early detection. A systematic search of research articles was conducted in four databases (PubMed, Scopus, Web of Science, and ScienceDirect) independently by two researchers. Publications retrieved in the independent search were mixed and imported into a single EndNote X8. The extraction of characteristics from the selected studies were carried out step by step by two independent researcher groups Abayeneh Girma and Fentaye Kassawmar and Yeshiwas Kassa and Yeshwas Asrat using a standardized data extraction format in Microsoft Excel 2021. Finally, the extracted data were combined and clearly presented in the table with the key information and findings. Inconsistencies between reviewers were resolved by discussion, and articles were included after consensus was reached. Totally, 2740 articles were retrieved, and 69 TB nanoparticle (NP)-based assays have fulfilled the inclusion criteria and included in this systematic review. The proposed platforms share the characteristics of accuracy, affordability, and swift time-to-result. Nanodiagnostics for TB now cover all clinical presentations of the disease, including active, drug-resistant, HIV-related, latent, and extrapulmonary TB. These advancements not only enhance the diagnostic landscape but also facilitate timely and effective treatment strategies, ultimately aiming to reduce the burden of TB worldwide. This review summarizes state-of-the-art knowledge of TB nanodiagnostics for the last 18 years. For fabrication concepts, detection strategies, and clinical performance, special consideration is given using various clinical specimens, and the suitability of TB nanodiagnostics for optimal MTB testing is evaluated. TB nanodiagnostics present a promising solution for meeting the stringent demands to end the TB epidemic by 2030.

## 1. Introduction

Tuberculosis (TB) is an infectious disease that primarily targets the lungs but can also affects the lymph nodes, pleura, brain, bones, and joints, among other organs [1]. Worldwide, TB remains the second deadliest disease despite anti-TB medication use [2]. According to the Global Tuberculosis Report 2023 of the World Health Organization (WHO), 1.3 million people died of TB, and there were 10.6 million new cases worldwide in 2022 [3]. Ninety percent to 95% of this bacterium's infections remain latent globally [4], while >98% of reported cases are from developing countries [5]. Treatment and control of TB patients were done by directly observed therapy short course (DOTS) strategy [6]. The decrease in the global decline of TB cases, particularly in developing countries, is due to antibiotic resistance [7]. The persistent challenge of multidrug-resistant TB (MDR-TB), including resistance to both isoniazid and rifampicin, significantly impedes effective TB treatment [8].

According to the WHO, an estimated 410,000 people developed MDR/rifampicin-resistant (RR)-TB worldwide in 2022 [3]. Accurate, early, and rapid detection of TB and antibiotic resistance is pivotal for effective TB management, particularly for MDR isolates, as it enhances treatment outcomes and prevents spread [8, 9]. Identifying acid-fast bacilli (AFB) through microscopic examination of sputum smears is the conventional diagnostic method for TB, but it fails to detect 50% of infected individuals, although it was invented in the 1880s. Consequently, it failed to detect half of all TB infections [10, 11]. Culture remains the gold standard for *Mycobacterium tuberculosis* (MTB) detection, although it takes over 6 weeks [12, 13]. Employing advanced molecular techniques like PCR, multiplex PCR, real-time PCR, loop-mediated isothermal amplification (LAMP), IS6110 restriction fragment length polymorphism (RFLP), line probe assays, PCR microarrays, allele-specific PCR, and genotype *M. tuberculosis* complex (MTBC) sequencing significantly decreases the time required for detection. They are considered rapid diagnostic systems for MTB DNA in clinical samples. These techniques are based on the design of primers and probes, but it needs high cost equipment's and materials with technical expert personnel [14]. Thus, nanotechnology-based diagnostics, including probe-based nanoparticles (NPs), offer economically feasible alternatives to high-cost equipment and expert personnel required for primer- and probe-based techniques and possess potential in detecting MTB, MTBC, and antibiotic resistance mutations, such as isoniazid and rifampicin resistance [15, 16]. NPs have high stability, ease of reactivity with biomolecules, and unique optical and catalytic properties, which make it as a crucial diagnostic tool for identifying MTB [17]. Thus, they can circumvent the limitations of molecular diagnostics [18]. Furthermore, these aspects of NPs effectively determine the design and creation of diverse nanodevices and diagnostic kits [19]. The current review provided a recent overview of the diagnostic value of NPs combined with other methods for TB detection.

## 2. Materials and Methods

### 2.1. TB Diagnostics Needs and Formulation of Research Questions

As clearly presented in Ghiasi and colleagues study [20], the most crucial research priorities in TB diagnosis for quick detection include developing (i) a sputum-free, swift test that displace sputum smear microscopy; (ii) a portable non-sputum biomarker test universally applicable for all types of TB; (iii) a rapid triage test for frontline healthcare providers to identify suspected cases of TB; and (iv) a microscopic-level drug susceptibility diagnostic test.

The aim of this systematic review was to determine “What are the NP-based assays that are used for the rapid detection of TB?” The problem was developed while examining the NP detection potential. Because of their diverse detection significance in diverse fields, the current study focused on examining the TB detection potential of NPs. In addition to this, we are interested in examining whether NP-based assays offer a viable alternative to existing TB detection methods for decreasing the hazards of TB or whether they can serve as an absolute option by replacing the conventional and molecular TB diagnostic methods.

### 2.2. Search Strategy

A systematic search of research articles was conducted in four databases (PubMed, Scopus, Web of Science, and ScienceDirect) independently by two researchers. The reference lists of the articles were checked manually to identify studies that were not accessed in online databases. The articles were searched using the following keywords extracted from the title, abstract, and keywords in combination or separately using Boolean operators (“OR” or “AND”): “nanoparticles,” “detection,” “diagnosis,” “tuberculosis,” “mycobacterium,” “*Mycobacterium tuberculosis*,” “MTB,” “*Pulmonary tuberculosis*,” “PTB,” “*Mycobacterium tuberculosis* complex,” and “MTBC.” The search strategy was conducted from August to October 2024.

### 2.3. Eligibility Criteria

The inclusion criteria were as follows: (i) original articles on NPs that address the detection of TB; (ii) an in vitro study design; (iii) only TB causative agent; and (iv) 2006–2023 studies and reported in English and published and available online. The exclusion criteria were as follows: (1) reported antimicrobial activities of NPs against TB or not linked to detection-dependent results; (2) other pathogens (other bacteria, fungi, parasites, or viruses); (3) studies not peer-reviewed and published in reputable journals; (4) reviews, case reports, correspondence, proceedings, and letters to the editor; (5) confusing or unclear analysis results or insufficient information; and (6) in vivo studies.

### 2.4. Study Selection

Publications retrieved in the independent search were mixed and imported into a single EndNote X8 (Thomson Reuters, USA) software. After excluding duplications, the titles and/or abstracts of the articles were attentively screened by the three research teams (Fentaye Kassawmar, Yeshiwas Kassa, and Yeshwas Asrat). Discrepancies between the reviewers were resolved through discussion or the fourth researcher (Abayeneh Girma) judged differences in the included literature after crosschecking. A full-text appraisal was conducted on articles that met our research question criteria and were considered sufficiently valid.

### 2.5. Data Extraction

Data from studies meeting the required criteria were extracted and recorded in an Excel spreadsheet. The information extracted from the data included NP type, sample type, target, signal detection method, clinical performance versus reference method, lower detection limit, and references which were extracted from the eligible studies (Tables 1 and 2). The extraction of characteristics from the selected studies was carried out step by step by two independent groups Abayeneh Girma and Fentaye Kassawmar and Yeshiwas Kassa and Yeshwas Asrat using a standardized data extraction format in Microsoft Excel 2021. Finally, the extracted data were combined and clearly presented in the table with the key information and findings. Inconsistencies between reviewers were resolved by discussion, and articles were included after consensus was reached.

## 3. Results

### 3.1. Outcome of the Search

**Figure 1:**
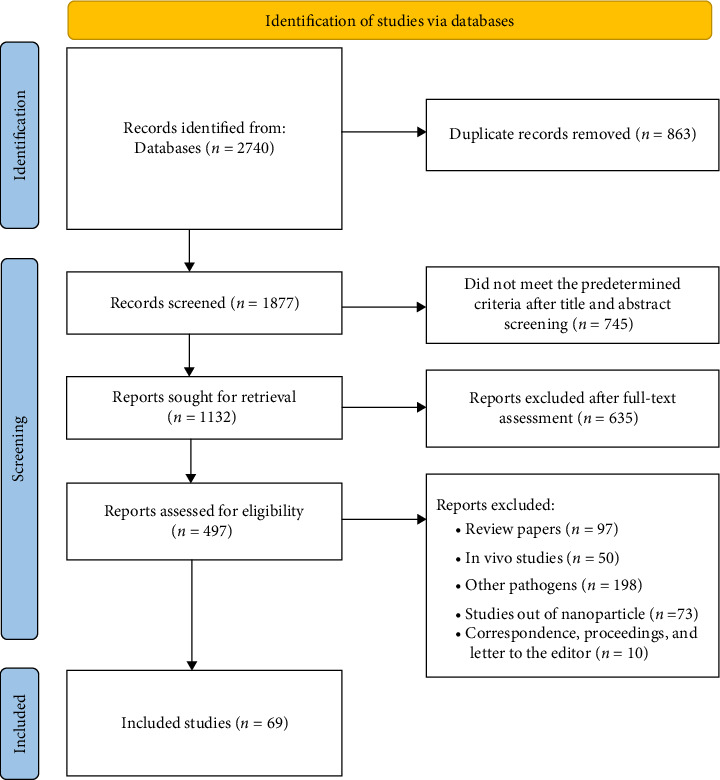
Flow diagram summarizing the selection of eligible studies.

Our prespecified retrieval strategy revealed 2740 relevant articles; 863 articles were excluded because of duplicates; 525 articles were excluded based on their incompatible titles and abstracts with the prespecified intervention. Another 635 studies were excluded after the full-text evaluation. Furthermore, a total of 428 articles were removed due to review articles (*n* = 97); in vivo studies (*n* = 50); other pathogens (*n* = 198); out-of-NP studies (*n* = 73); and correspondence, proceedings, and letters to the editor (*n* = 10). In the end, 69 articles were identified as meeting the requirements and were subsequently incorporated into the systematic review (Figure 1). Around 45 and 24 articles used NP-based DNA and immunodiagnostics approaches for the timely detection of TB, respectively.

### 3.2. General Characteristics of the Included Studies

**Figure 2:**
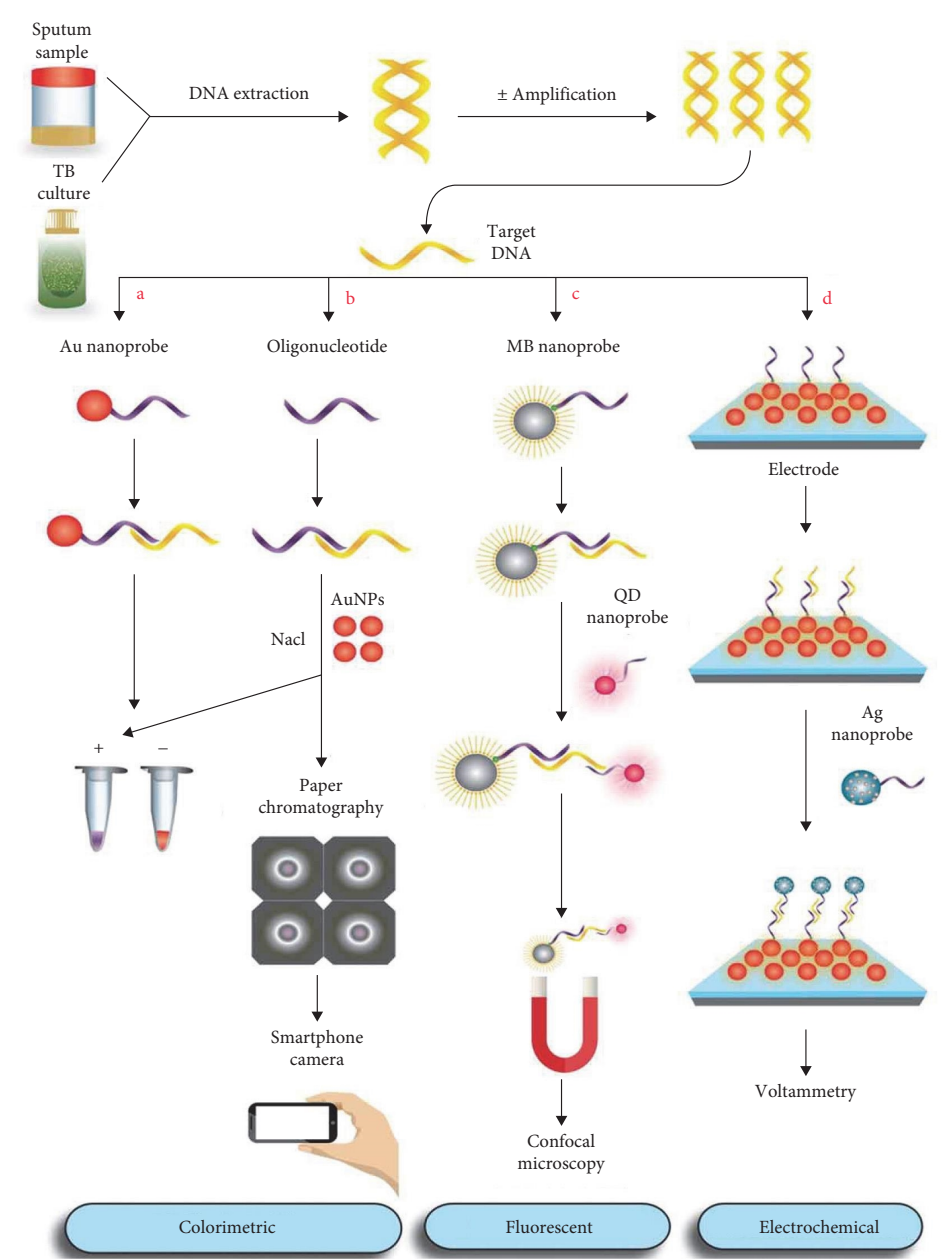
Some picked nanoparticle-based detection methods applied for TB DNA detection. Reproduced with permission [16]. (a) In the cross-linking assay, TB-specific gold nanoprobes hybridize with target DNA in PCR products. As a result of probe hybridization, Au nanoprobe aggregates change color from red to blue, indicating a positive response. For negative samples, red color is retained when using unmodified gold nanoparticles (AuNPs) for the detection of TB [32]. (b) Unmodified AuNPs are added to the test solution following the addition of salt. Upon detection of target DNA (TB positive), salt cations induce the aggregation of AuNPs and subsequently result in a color change from red to blue due to the hybridization of oligonucleotides to target DNA. For negative samples, the AuNP color remains unchanged due to electrostatic adsorption of oligonucleotides onto their surface. A smartphone camera can be utilized to digitize colorimetric responses. The test solution, with the aid of a solid wax as a hydrophobic barrier, is applied to the chromatography paper after the color has developed in order to concentrate it. Through red–green–blue (RGB) analysis, a smartphone application processes and examines a captured image [34]. (c) A TB-specific nanoprobe attached to magnetic beads coated with streptavidin is introduced to genomic DNA for hybridization purposes. A second probe specific to TB, attached to CdSe quantum dots, is employed for sandwich hybridization following the magnetic separation of the hybridization complex. The conjugate is separated from the solution and then transferred to confocal microscopy for direct visualization of fluorescence [56], while (d) streptavidin-coated AuNPs (AuNPs-ST) are immobilized and functionalized on the electrode surface with biotinylated capture probes specific to TB via biotin–streptavidin affinity. The target DNA is added to initiate hybridization. A signal probe labeled with luminol-modified silver nanoparticles (AgNPs) is introduced for sandwich hybridization. Voltammetry detects the electrochemical signal generated by the double-step hybridization process [61].

**Figure 3:**
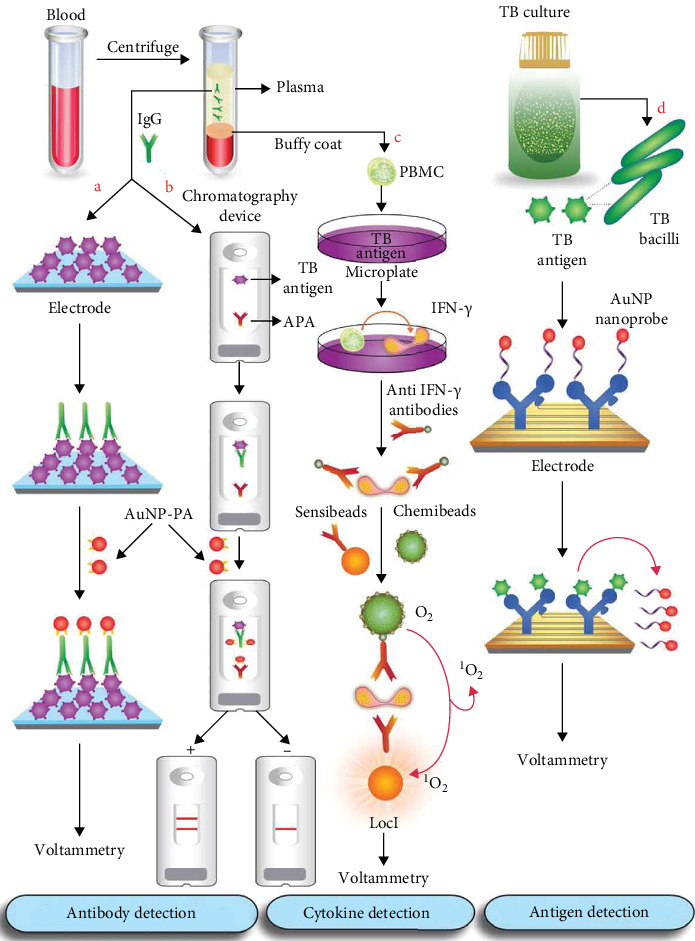
Some picked nano-immunodiagnostics applied for TB DNA detection. Reproduced with permission [16]. (a) The carbon electrode surface is coated with lipoarabinomannan (LAM) antigen. Adding anti-LAM-IgG results in the formation of an antigen–antibody complex. The helicases I and II of SPA interact with the Fc region of anti-LAM (IgG), which is bound to gold nanoparticle (AuNP) modified with protein A (AuNP-PA). AuNPs have been successfully electro-oxidized for use in DPV detection [66], while (b) an immunochromatographic test device specifically targets TB antibody, interacting with the 38-kDa antigen in the nitrocellulose membrane on the test line. Protein A-coated gold nanoparticles (AuNP-PA) pass through the membrane to interact with antigen–antibody complexes, creating a red line. Once bound with anti-protein A (APA), AuNP-PA conjugates move to the control line and generate a second red line. Two red lines indicate a positive result, while one red line signifies a negative result [67]. (c) In a luminescent oxygen channeling immunoassay (LOCI) test, peripheral blood mononuclear cells (PBMCs) presensitized to TB are placed in a microwell plate with ESAT-6 and CFA10 antigens to elicit IFN-γ release. An anti-IFN-γ antibody labeled with biotin is introduced for immune complex formation. The complex is reacted with streptavidin-coated chemibeads via biotin–streptavidin affinity and with anti-IFN-γ antibody-labeled sensibeads via antigen–antibody reaction. When exposed to light, chemibeads generate singlet oxygen, causing a chemiluminescent reaction with sensibeads [88]. (d) Ninety-six-hour culture filtrates are added to the uniquely designed electrode, which is coated with gold, connected with a series piezoelectric quartz crystal (SPQC), and functionalized with a dual-target aptamer-attached TB DNA-specific Au nanoprobe. The Au-probe is released due to a stronger antigen–aptamer bond between ESAT-6 and CFP10 antigens. The electrical frequency changes are digitally detected and processed [69].

As presented in Tables 1 and 2 and Figures 2 and 3, all included studies were conducted on NPs (NP-based assays and nano-immunodiagnostics), showed the detection potential of TB, and demonstrated different clinical performances in different samples. The majority of the included studies used gold NPs (AuNPs) for the detection of TB, while others used other types of NPs like silver (Ag), magnetic, silica, quantum dots (QDs), nanodimonds, and nanocomposites, among others. The samples used for diagnosis include clinical isolates, reference strains, sputum, blood, or synthetic DNA from TB, whereas for nano-immunodiagnostics, human serum, sputum, saliva, urine, blood, reference isolates, culture filtrates, or synthetic antibodies are used. For TB diagnosis, most studies target DNA or antigens. TB was detected using colorimetric, electrochemical, fluorescent, chemiluminescent, and nuclear magnetic resonance. The sensitivity and specificity, lower detection limits, and performance measurements vary from study to study. The turnaround time varied from 5 s to 96.3 h.

## 4. Discussion

Rapid TB detection is crucial for reducing the risk of TB infection and antibiotic resistance. Detecting the virulent TB strain swiftly, efficiently, and economically is a crucial aspect of the worldwide battle against TB. Therefore, developing other detection methods of TB like nanobased assays is required and is useful as a guide for both governmental and nongovernmental policymakers and stakeholders to control and support the global End TB strategy by 2030.

### 4.1. NP-Based Assays

#### 4.1.1. DNA Detection Approach

##### 4.1.1.1. AuNPs

In 2006, Baptista et al. [21] created the first MTB detection assay employing spherical AuNPs for DNA recognition (Table 1). AuNPs were functionalized with thiol-modified oligonucleotides specific to MTB RNA polymerase subunit gene sequence for colorimetric detection of positive MTB specimens without cross-linking. At an absorbance peak of 526 nm due to surface plasmon resonance (SPR), the red color of Au nanoprobescolloid shifts to blue as a result of their aggregation in high NaCl concentration. At high salt concentrations, complementary MTB sequences prevent Au nanoprobes from aggregating posthybridization. The solution retains its red color. Researchers extracted DNA from clinical specimens for their study. The Au nanoprobe solution was added, and denaturation and annealing conditions were optimized following DNA amplification by PCR. Fifteen minutes after adding NaCl, the colorimetric result was achieved either by visual observation or spectrophotometric measurements.

Using three Au nanoprobes, Costa et al. [22] distinguished MTBC members, including MTB, from *M*. *bovis* in molecular discrimination of various mycobacteria strains. Another study [25] was carried out to assess the accuracy of detecting RR MTB in sputum isolates. Seven nanoprobes were created: one for the rpoB gene found in all MTBC variants and six targeting three frequent resistance to rifampicin's (RIF's) resistance mutations (equally distributing toward mutated and wild-type alleles).

The cross-linking colorimetric assay for MTB detection was used by Soo et al. [23]. When Au nanoprobes hybridize to target DNA, this aggregation of Au nanoprobe/target complexes occurs, causing a red-to-blue color shift, indicative of a positive result. Through nested PCR, we targeted IS6110 in MTBC and Rv3618 in MTB using two primer pairs. Au nanoprobes specific to IS6110 and Rv3618 were added to the amplicon for colorimetric detection.

Hussain et al. [32] employed unaltered AuNPs for colorimetric identification of MTB. By forgoing the prior functionalization of AuNPs with oligonucleotides, this strategy saved both time and cost. Nitrogenous bases of free oligonucleotides bind electrostatically to the negatively charged AuNPs in the presence of salt. The red color of the AuNP colloid is maintained by adsorption. The presence of target DNA triggers the aggregation of AuNPs through hybridization of oligonucleotides to complementary sequences, leaving the AuNPs exposed to salt cations.  Figure 2a shows the color change from red to blue. Tsai et al. [34] optimized the assay by combining a paper-based platform and a smartphone camera for color detection. After color development, the AuNP solution was applied to chromatography paper with wax to confine the color within a circle. The red–green–blue (RGB) smartphone software captured and analyzed the image (Figure 2b).

Veigas et al. [35] optimized the colorimetric detection of sputum isolates, including various mycobacterial strains, using LAMP instead of conventional PCR. Three Au nanoprobes were designed to distinguish MBTC members, wild-type RIF, and mutant-type RIF, specifically targeting the rpoB nucleotide. Upon completion of DNA extraction and isothermal amplification, Au nanoprobes were introduced, with salt then added to trigger aggregation and color change.

The research group integrated paper chromatography into their analysis method for color detection. After attachment of Au nanoprobes to target DNA, the complexes were deposited on a salt-impregnated wax-printed multiwell plate. Fifty minutes later, the smartphone captured an image and analyzed it using RGB software after the photo's colors had developed against a white paper background. The paper platform was used to identify RIF-resistant strains. The proposed platform failed to generate a vibrant enough color to clearly distinguish positive from negative outcomes. To accommodate the microplate's capacity, a smaller volume of Au nanoprobe was used in the test (5 mL) versus the initial version's larger volume (30 mL) [35].

Silva et al. [26] constructed a biosensor combining a nearly spectral flat silicon nanophoto detector (520–630 nm range) with a cost-effective, double-wavelength red, green, blue, and amber (RGBA)-LED light source, as well as a printed circuit board for signal (PCB). The RGBA-LED illumination triggers the test solution (AuNPs/test DNA/salt), and then the resulting light passes through and is then converted to photoelectric signals by the nanophoto detector; this PCB circuitry processes and records the data. The generated waves undergo digital transformation and are sent to a computer for processing into numerical results through a USB connection by user-friendly software. The gyrB gene sequences of MTBC, MTB, and *M*. *bovis* were targeted through integration of three Au nanoprobes. The proposed biosensor recognized different mycobacterial strains post-PCR.

Thiruppathiraja et al. [27] made an AuNP-coated indium tin oxide (ITO) electrode for identifying MTB genomic DNA directly from sputum samples of TB suspects. Two probes, one for capturing DNA using a specific sequence and the other for detection, are marked with alkaline phosphatase enzyme (ALP). The ITO electrode was functionalized with a capture probe and then immersed in hybridization buffer containing sonicated target genomic DNA to form double-strand DNA. The ITO electrode was immersed in the Au-nanoprobe labeled with ALP for hybridization sandwich development. Electric signals from ALP catalytic reactions during hybridization events were recorded using differential pulse voltammetry (DPV).

Liu et al. [37] employed reduced graphene oxide (rGO) and polyalanine (PANI) for an electrochemical method, taking advantage of rGO's thermal conductivity, large surface area, and PANI's rapid electron transfer and electrochemical activity. The novel electrobiosensor employed an rGO-AuNP nanocomposite matrix and utilized an Au-PANI nanocomposite-labeled DNA probe as the direct electron mediator. IS6110-targeting probes, one with biotin capture ability and the other labeled with Au-PANI nanocomposites, were employed in the study. After adding capture probes onto the rGO-AuNP nanocomposite surface, the electrode was immersed in the target DNA for 2 h, and then the signal probe was added, followed by a 2-h incubation. The electrochemical measurements were obtained using a conventional three-electrode system. The peak current was reduced twice upon applying a positive sample: once upon the capture probe binding to the rGO-AuNP electrode surface. Upon hybridization, the target DNA binds to the capture probe.

Torres-Chavolla and Alocilja [28] developed an electrochemical DNA biosensor for MTB detection using amine-terminated magnetic particles (MPs) and dextrin-coated AuNPs. Using IS6110-specific probes, one probe was functionalized to capture MPs, while another was altered to act as AuNP reporter. After amplification of target DNA using helicase-dependent isothermal amplification (tHDA), the MP-capture probe was added, followed by the Au-reporter probe, which was hybridized, and the sandwich complex (MP-target-AuNP) formed was transferred to a screen-printed carbon electrode (SPCE) and electric measurements recorded using DPV.

Ng et al. [38] designed another electrochemical biosensor. The proposed biosensor stands out due to its innovative application of a rapid MTB DNA extraction method (modified SPR imaging [SPRI] technique) from sputum isolates, isothermal amplification via recombinase polymerase amplification (RPA) targeting the RpoB gene of MTB, and biotinylated amplicon production through RPA supplementation with biotin-deoxyuridine triphosphates (dUTPs). Biotinylated products bound first to the streptavidin (SA)-coated electrode surface. SA-functionalized AuNPs bound with free biotin of RPA products. An AuNP oxidation signal was detected via DPV measurement in the end.

Xiang et al. [36] developed a real-time and multiplex SPR biosensor for the detection of five RIF-resistant MTB mutations. Five unlabeled capture probes and five linear padlock probes (PLPs), in addition to an MTB-specific Au nanoprobe, were integrated for the detection of mutant alleles amplified by the ligation-rolling circle amplification (L-RCA) method. The linear PLP design features three binding sites: a target site recognizing point mutations and enabling circularization, a tag site hybridizing with capture probes attached to the Au-coated sensor chip and serving as a primer for L-RCA, and a general site interacting with Au nanoprobes. A change in SPR angle, detected by the sensor, is the result of a successful sense/antisense interaction. The signal undergoes a 12-fold amplification before detection due to the features of RCA and AuNPs.

The SPR biosensor [39] was enhanced to offer real-time label-free detection via the optimization of the group, with RCA-cleavage reaction utilized for amplification of the target DNA. Performing the cleavage reaction with a restriction enzyme generated small single-strand DNA pieces for direct hybridization with probes immobilized on the chip surface. AgNPs, without label, were aggregated on the chip to amplify the SPR signal. The PLP consists of a distinctive code sequence, two target-specific sequences, and an enzyme-cutting site. The SPR biosensor identified MTB and *M*. *bovis* genomic DNAs simultaneously.

##### 4.1.1.2. Magnetic NPs (MNPs)

Liong et al. [52] developed a microfluidic device capable of executing all detection procedures following DNA extraction from clinical isolates. The DNA was amplified using PCR with rpoB and fadeE15 sequences and then labeled magnetic nanoprobes, and capture beads were used for magnetic capture. NMR measurements detected the signal.

Engström et al. [53] devised a novel system for detecting RIF mutations in the rpoB gene using SA-tagged magnetic nanobeads functionalized with biotin-labeled oligonucleotides. Eleven PLPs were added, including one for MTBC detection of the 16S-23S ITS region, another for wild allele discrimination, and a mix of nine for identifying common mutant alleles in the RIF resistance-determining region (RRDR)-rpoB gene. Selected PLPs were added after the DNA was extracted from clinical isolates. By hybridizing to the target, circularization of PLPs was accomplished, followed by their amplification via two rounds of RCA to boost sensitivity. Oligonucleotide-tagged beads were introduced to anneal to the PLP backbone's hybridization site in the RCA products. Applying the Brownian relaxation principle, the detection strategy relies on the increase in hydrodynamic size of magnetic beads (MB) upon hybridization with the target sequence, resulting in a decrease in relaxation frequency. A portable AC susceptometer was used to detect the signal.

##### 4.1.1.3. QDs

Using a sandwich-form biosensor design based on fluorescence resonance energy transfer (FRET) strategy, Shojaei et al. [57] fabricated the sensor using cadmium telluride QDs (CdTe QDs) as a fluorescent donor. Two probes, one conjugated to 6-nm CdTe QDs and the other to AuNPs, were designed to target the esat-6 region. The overlapping emission and absorption spectra of CdTe QDs and AuNPs enable their dual use as fluorescent donors and acceptors. The TB + isolates' DNA was extracted and amplified using PCR. Nanoprobes were added to hybridize with their complementary targets. Fivefold enhanced fluorescence was detected by a spectrophotometer upon quenching of the fluorescent moiety of CdTe QDs by AuNPs through FRET.

Gazouli et al. [56] detected MTB and *M*. *avium* subsp. *paratuberculosis* (MAP) in bronchoalveolar lavage (BAL), fecal, and paraffin-fixed tissue samples using SA-coated CdSe QDs. The platform utilized five probes: three SA-coated MB probes recognizing the *Mycobacterium* genus via the 23S rRNA locus and two biotinylated detection probes associated with SA-coated CdSe QDs, each targeting IS6110 and IS900 loci to differentiate MTB and MAP. Primarily, MB probes were directly hybridized to extracted gDNA. Next, the magnetic device was used to separate the MBs probed with DNA targets from the solution. Then, QD probes were annealed to their complementary targets on the complex. Eventually, the conjugate of the MB probe, DNA target, and QD probe was then separated and subjected to UV apparatus or confocal microscopy for fluorescent signal detection (as shown in Figure 2c).

Yan et al. [58] developed a multiplex assay for MTB and HIV detection in serum using PowerVision technology, which immobilizes abundant QDs through a dendrimer enzyme-linked polymer. Two oligonucleotides, one specific to TB (Au-sTB) and the other to HIV (Au-sHIV), were assembled on PV-lead sulfide (PbS) and PV-cadmium sulfide (CdS) conjugates, respectively, by means of utilizing the different anodic stripping voltammetry peaks of their corresponding CdS and PbS QDs. The Au-sTB/PV-PbS and Au-sHIV/PV-CdS complexes continued to function as signal tracers. Two capture probes, each with a specific thiol-modified oligonucleotide (complementary with TB [cTB] and complementary with HIV [cHIV], respectively), were designed and immobilized onto AuNP-coated MBs (GMPs). The net product comprises GMPs/cTB and GMPs/cHIV conjugate. With the addition of target DNA, dual hybridization occurred between the DNA, signal tracers, and capture probes. The net hybridization product was a combination of signal tracer, target DNA, or capture probe. Anodic stripping voltammetry yielded the signal detection.

##### 4.1.1.4. Other NPs

He et al. [61] functionalized AgNPs with SA-coated AuNPs and then immobilized them on the ITO electrode surface to assemble biotinylated-capture probes specific to TB. The addition of target DNA triggers the hybridization process. A signal probe tagged with luminol-labeled AgNPs was introduced for sandwich hybridization. The electrochemiluminescence (ECL) signal was generated and detected by electrochemical impedance spectroscopy (Figure 2d) through a double-step hybridization process. Wang et al. [62] integrated SA NPs into their hybridization signal amplification method (HSAM) system, which allowed for MTB detection in sputum samples using an amplified fluorescence signal, eliminating the need for DNA amplification. Two IS6110-specific probes, each composed of a biotinylated capture probe attached to SA-coated MBs and a bis-biotinylated detection probe interfaced with SA NPs, were employed. Following DNA extraction from clinical isolates, capture hybridization was performed using a probe. The probe was next added to the probe/target complex for the immobilization of SA-coated NPs via hybridization. A biotinylated-fluorescein isothiocyanate (FITC) signal was detected through fluorescence microscopy. The three-dimensional SA NPs' ability to bind fluorescein multiple times and utilize biotin/SA affinity resulted in a thousand-fold signal amplification.

Rare earth lanthanide elements make up upconverting NPs (UCNPs), found in their crystalline structure. Hwang et al. [63] developed a FRET-based biosensor for the detection of IS6110 sequences in amplified MTB DNA obtained from sputum samples. SA-coated UCNPs were linked to biotinylated PCR products. The fluorescent dye (SYTOX Orange) intercalated into the labeled amplicons. Due to the overlap of the maximum excitation wavelength of SYTOX Orange (547 nm) and the emission wavelength of UCNPs (543 nm) from a 980-nm NIR laser, the energy of UCNPs was transferred to SYTOX Orange, thereby diminishing UCNPs' fluorescence intensity, which was measured using a fiber optic spectrometer.

#### 4.1.2. Immunoassays and Immunodiagnostics

##### 4.1.2.1. AuNPs

Wang et al. [66] developed an electrochemical serodiagnostic tool for detecting TB anti-lipoarabinomannan (LAM) antibodies (as shown in Table 2). AuNPs, labeled with staphylococcal protein A, served as an electrochemical tag. The carbon electrode was first coated with LAM, followed by the addition of a solution containing anti-LAM IgG. The immune complex formed between LAM and anti-LAM IgG was then attached to Au-PA through the interaction between PA helicases and the Fc region. AuNPs were finally electro-oxidized for DPV detection (Figure 3a). The assay had a detection limit of 5.3 ng antibody per milliliter of serum. The serum antibody level of TB patients surpassing 10,000 ng/mL has clinical applicability potential.

A flow cytometry (FCM)-based immune-chromatographic assay for detecting MTB in whole-blood and serum samples was developed by Mdluli et al. [67], targeting an anti-38-kDa monoclonal antibody. The assay involved dispensing MTB fusion 38-kDa antigen and anti-protein A antibody onto a nitrocellulose membrane as test and control lines, respectively. The AuNPs were immobilized on the membrane through protein A conjugate pads after being modified with protein A to form the AuNP-PA conjugate. If a blood sample tests positive, the target antibody will bind to the MTB antigen, resulting in antigen/antibody complex formation. The line turned red in the intricate setup. The AuNP–PA complex moved to the line of anti-protein A antibodies, forming an additional antigen/antibody complex that showed the red control line (Figure 3b).

An electrochemical immunosensor for MTB detection in serum was developed by Zhang et al. [68] using interferon-γ (IFN-γ). To provide a surface for immobilizing INF-γ-specific capture antibody (Ab1), positively charged poly(diallyl dimethyl ammonium chloride) (PDDA)-coated ITO electrodes (due to electrostatic interaction) have been used to assemble negatively charged AuNPs. The horseradish peroxidase (HRP)-Ab2-AuNPs conjugate, made by labeling another batch of AuNPs with HRP-bound INF-γ-specific antibodies (Ab2), served as the signal tag. The immunosensor was placed in an electrochemical cell with hydroquinone (HQ) and hydrogen peroxide (H_2_O_2_). The electrochemical signal was detected via DPV after amplification by AuNPs and HRP following HQ oxidation by H_2_O_2_.

Three sensors for MTB applications were innovated with the use of series piezoelectric quartz crystals (SPQC) in their design. According to the Sauerbrey equation, small weight variations on a crystal electrode cause oscillation frequency shifts. These platforms are economical but unspecific toward pathogenic MTB strains. The research group [69] enhanced test performance by designing a dual-target aptamer-coated Au nanoprobe and immobilizing it on Au-coated/SPQC-connected electrodes. Upon adding the TB culture filtrate, the aptamer specifically identified and detached the Au nanoprobe from MTB antigens via the antigen/aptamer reaction, with a stronger interaction than that between the aptamer and complementary nucleic acid. The SPQC sensor (Figure 2d) measures the change in electric properties.

##### 4.1.2.2. MNPs

Lee's team [77] synthesized MNPs with significantly large Fe cores and thin ferrite shells (CBs) for maximized transverse reflexivity (r2) per particle. CBs were coated with anti-BCG monoclonal antibodies to facilitate bacterial capture. Bacteria were concentrated, and the analyte purified using a chip-based filter system before detection with NMR spectroscopy. The specimen is first incubated with CB-AB to magnetically capture and concentrate bacteria. Captured bacteria were retained in the microfluidic chamber by dispersing excess CB through repeated injections of washing buffer. NMR measurements were taken on bacteria that were resuspended through membrane backwashing.

Lee et al. [78] have developed a new magnetic resonance imaging (MRI)-based tool using super-paramagnetic iron oxide (SPIO)-anti-MTB surface antibody (TBsAB) conjugates to diagnose extrapulmonary TB. SPIO-TBsAB was inoculated with *M. bovis*, which had been previously cultivated on Middlebrook media and preincubated with activated THP-1 cells. The T2-weighted MR image later confirmed probe-target recognition through decreased signal intensity.

##### 4.1.2.3. Silica NPs

Qin and coworkers [79] used RuBpy-doped silica NPs with high luminescence and photostability for a rapid immunodiagnostic assay for TB detection, wherein rabbit anti-MTB antibody captured MTB. By immunological reaction, the antigen/antibody complexes were adsorbed onto silica NPs functionalized with protein A, resulting in a detectable fluorescent signal observable via fluorescence and confocal microscopies. The assay successfully identified MTB in mixed bacterial samples and TB-spiked sputum samples. The fluorescent signal from the test was five times better than the standard FITC method as evaluated the clinical efficacy of the assay by Ekrami et al. [81].

Two-color FCM was employed with SYBER Green-1 staining to further enhance the detection of antigen/antibody complexes [80]. The modification prohibited false-positive results from occurring due to spontaneous NP aggregations and nonspecific binding to sample debris.

##### 4.1.2.4. Other NPs

In positive mycobacteria growth indicator tube (MGIT) culture for MTBC detection, detonation nanodiamond (DND) was employed [87] as a matrix-assisted laser desorption ionization time-of-flight mass spectrometry (MALDI-TOF MS) sample pretreatment agent (4–5 nm in size). Adding 5-nm DND to 1 mL of MGIT broth increases the broth's ability to capture and concentrate CFP-10 for enhanced MALDI-TOF MS sensitivity in MTBC strain identification. After DND treatment, MALDI-TOF MS examination of a positive MGIT broth filtrate revealed a 50-fold increase in peak intensity. The modified DND method had a lower detection limit than the standard method. Forty-two MIGIT samples with positive results were evaluated using the updated assay, and their findings were confirmed with traditional culture-based biochemical techniques.

Liandris et al. [86] presented an affordable and uncomplicated immune-diagnostic platform using SA-coated CdSe QDs and MBs that were functionalized with biotinylated anti-MTB antibodies. Bacteria labeled with MBs and anti-AB polyclonal antibodies were magnetically extracted from the solution and then tagged with anti-heparin-binding hemagglutinin, a biotinylated antimouse antibody, and SA-conjugated QDs. The fluorescence signal was observed visually or measured using a fluorometer.

A luminescent oxygen channeling immunoassay (LOCI) was developed for detecting LTB in blood samples [88]. The sandwich immunoassay utilized chemiluminescent signal detection to quantify IFN-γ levels through the use of acceptor sensibeads labeled with anti-IFN-γ monoclonal antibodies and donor chemibeads coated with SA. Foremost, 5 mL of venous blood was used to isolate peripheral blood mononuclear cells (PBMCs). Next, 96-microwell plates were seeded with two PBMCs each, one receiving esat-6 and the other cfp-10 o MTB-specific antigens. Third, a biotin-labeled IFN-γ antibody was added to each well to bind with IFN-γ (released upon antigen recognition by presensitized T cells in positive samples), forming IFN-γ-antibody/biotin complexes. After adding anti-IFN-γ monoclonal antibody-labeled acceptor sensibeads, the complexes were separated to create a sandwich complex. Lastly, SA-coated donor chemibeads and an immunoluminescent reagent were employed to capture the complex and detect signals (Figure 3c).

### 4.2. The Common Properties, Advantages, and Limitations of Each NP Type in the Detection of TB

Several characteristics, which enable TB diagnostic purposes, exist in almost all of these NPs. The large effective surface-to-volume ratio allows NPs to accommodate biomolecule attachment points such as antibodies, aptamers, and DNA, which improve TB test capabilities. The NPs have exceptional bioconjugation attributes that allow them to accept TB-specific binding molecules for targeted detection of *M. tuberculosis* or its biomarkers. Their tinier dimensions make possible precise molecular-level recognition used for detecting very limited amounts of TB pathogens. These NPs demonstrate excellent abilities for integration into point-of-care (POC) diagnostic tools which enables their use in portable compact systems like lateral flow assays and biosensors and smartphone-based devices (Table 3).

AuNPs are the ones that brings numerous benefits to TB detection because they exhibit SPR and other optical features that generate visible color changes, which make them suitable for basic tests using lateral flow testing methods. Simple synthesis procedures of these NPs allow effective functionalization along with robust methods that combine TB-specific antigens or DNA or antibodies while removing the necessity for additional tagging methods. The materials present biocompatibility properties that keep them harmless for use in biological testing environments. Their utility has restrictions because they have limited capability to detect multiple markers simultaneously. Biochemical samples present two major challenges to color changes from sensors because these changes prove hard to quantify with basic methods without spectrophotometric tools and because they may produce unstable or hard-to-detect changes [41, 49, 90, 96].

The MNPs is the other that contain unique strengths as well as specific operational constraints. The main advantages of MNPs include (1) magnetic separation that utilizes external magnets to quickly extract TB biomarkers from intricate biological media like blood and sputum and (2) enhanced sensitivity from amplified magnetic biosensor detection at low TB concentrations, (3) large surface area availability to densely attach detection probes like TB-specific agents, and (4) compatibility with microfluidic systems vital for automated or semi-automated diagnostic devices. The limitations of MNPs include (1) agglomeration risks due to uncoated or unstable MPs and (2) external magnetic field necessity that complicates device design and (3) possible toxicity from iron particles generating ROS when not properly coated [91, 92, 97].

QDs are the third that offers both favorable aspects and technical constraints. The main benefits of TB detection through QDs entail (i) fluorescent signaling which provides strong and adjustable fluorescence for superior sensitivity along with multibiomarker analysis as well as (ii) persistent signal endurance that surpasses organic dye stability and (iii) simultaneous biomarker recognition facilitated by the size-based color emissions and (iv) high detection sensitivity that aids in identifying minimal bacterial counts or drug resistance elements. The utilization of QDs faces challenges due to their heavy metal-based composition such as cadmium because it introduces toxicity risks that may affect biological compatibility. The production methods for QDs involve elaborate procedures with committed techniques that ensure their dispersal and chemical stability in biological solutions. Producing QDs normally requires higher expenses than manufacturing either AuNPs or MNPs. Advanced fluorescence readers such as fluorometers or confocal microscopes serve for QD detection, but these sophisticated instruments restrict their deployment at POC testing sites especially in under-resourced settings [93–95].

In general, AuNPs (lateral flow assays for rapid sputum-based TB screening in field settings), MNPs (capture and enrichment of *M*. *tuberculosis* DNA/RNA for qPCR or biosensor analysis), and QDs (high-precision lab-based detection of TB proteins and drug-resistance markers in research settings or centralized labs) are crucial detection alternatives. Different NP types provide distinct advantages for detecting TB. AuNPs are fast and economical, but MNPs provide enhanced sensitivity for biosensing systems, while QDs offer high sensitivity and multiplexing except for toxicity risks and production expenses (Table 3).

### 4.3. Cost Comparison of NP-Based TB Diagnostics Versus Conventional Methods

A single TB detection sample analysis costs greatly based on which diagnostic procedure gets utilized. According to Hao and colleagues' systematic review [98], the average cost per TB test was 29.8 US$ for Xpert compared with 3.83 US$ for smear microscopy. Groessl et al. [99] studied on cost analysis of rapid diagnostics for drug-resistant TB and reported that the mean cost per sample without equipment or overhead was $23, $28, $33, and $41 for the microscopic observation drug susceptibility assay, MGIT, pyrosequencing, and line-probe assay, respectively. However, NP-based biosensors have estimated costs ranging from $0.10 to $0.50 per test according to the biosensor complexity and used materials [55, 100, 101]. The expenses of developing and acquiring equipment remain high in initial stages, but the unit prices are predicted to lower down when commercialization increases and production volumes grow. POC usable detection methods combined with quick (minutes) response time and high precision rate (sensitivity and specificity) represent the paramount features of these tests. Widespread implementation of diagnostic principles faces three main barriers today including regulatory barriers and assessment reliability problems and specialized equipment needs in particular conditions.

### 4.4. Limitations of This Review

The current study has certain limitations. The potential benefits of the process remain unrealized in clinical practice. While most published research stops at demonstrating conceptual proof, a few case–control studies are an exception. The accuracy of most assays has been overestimated due to the use of artificially spiked samples or isolated DNA extracts instead of real clinical samples. Several DNA-based assays involve an amplification step before detection, leading to increased costs and time. No assay for TB in children has been developed. According to global NTP managers' priority criteria, TB nanodiagnostics are potential candidates for POC testing.

## 5. Conclusions and Future Perspectives

TB continues to pose a significant public health concern in the face of contemporary scientific advancements. The WHO has strengthened her commitment to eradicate the global TB issue by 2035. In this study, 69 NP-based TB detection assays have been characterized, some partially and others fully, through DNA-based or immunological methods. The complexity of fabrication for proposed assays can differ based on NP processing, assay-related integrals, and signal detection methods (colorimetric, electrochemical, fluorescent, chemiluminescent, and nuclear magnetic resonance). NP-based assays integrating paper-based/smartphone platforms, microfluidics, and isothermal DNA amplification technology have been developed. These platforms aim for quick turnaround, precision, and affordability. The assessment of performance demonstrated an 80%–100% concordance with the reference methods. Results can be obtained within a 5-s to 96.3-h time frame. NP-based TB diagnostics have estimated costs ranging from $0.10 to $0.50 per test. For a comprehensive understanding of TB, numerous nanodiagnostic tools have been formulated to cover the active pulmonary phase, discrimination against non-TB mycobacteria and MTB complex members, drug-resistant MTB, and TB/HIV coinfection, as well as latent TB. Developing NP-based TB diagnostics with high sensitivity and accuracy, yet not needing sputum samples, is a promising future direction.

## Figures and Tables

**Table 1:** Appears to focus on DNA-based detection.

Nanoparticle type	Sample type	Target	Signal detection method	Clinical performance versus reference method	Lower detection limit	Turnaround time	Reference
Gold	Sputum, bronchial lavage, pleural effusion, urine, and blood (*n* = 73)	RpoB locus for MTB	Colorimetric by direct observation or spectrophotometry	100% sensitivity, 89% specificity compared to INNO-LiPA-Rif-TB	0.75 g	2 h	[21]
	Clinical isolates	gyrB locus for MTBC	Colorimetric by direct observation or spectrophotometer	100% concordance with PCR-RFLP	NA	15 min after PCR	[22]
	Clinical sputum specimens (*n* = 600) and mycobacterium ATCC reference strains (*n* = 23)	• IS6110 locus for MTBC • Rv3618 for MTB	Colorimetric	96.6% sensitivity and 98.9% specificity toward the detection of MTBC and a 94.7% sensitivity and 99.6% specificity for the detection of MTB as compared to culture	0.5 pmol	2 h after amplification	[23]
	MAP DNA in faces	16–23s ITS DNA region of MAP	Colorimetric by direct observation or spectrophotometer	87.5% sensitivity and 100% specificity concordance with qPCR	18.75 ng	NA	[24]
	Clinical isolates (*n* = 42)	rpoB locus for MTBC and MDRTB	Colorimetric by direct observation or spectrophotometer	83.3, 81% concordance for MTBC and MDRTB compared to INNO-LiPA Rif-TB	75 fmol	15 min after PCR	[25]
	Reference and clinical isolates	gyrB locus for MTBC, MTB, and *M*. *bovis*	MATLAB (after signal processing by PCB and ADC)	100% concordance with culture	50 nM	Less than 30 min after PCR	[26]
	Genomic DNA of MTB and sputum samples (*n* = 43)	*Mycobacterium* sp. genomic DNA	Electrochemical by DPV	NA	1.25 ng/mL genomic DNA	NA	[27]
	Synthetic target ssDNA	IS6110 locus for MTBC	Electrochemical by DPV	NA	0.1 ng/µL	6 h after DNA extraction	[28]
	Synthetic target ssDNA	IS6110 locus for MTB	EIS and ECL	NA	3.3 × 10^−16^ M	NA	[29]
	Synthetic target ssDNA	rpoB locus for MTBC	Colorimetric	NA	NA	NA	[30]
	Sputum samples (10 samples as positive culture, 8 samples as negative culture, and H37RVKK11 20 as standard strain)	IS6110 locus for MTB	Quartz crystal microbalance assay	100% sensitivity, 100% specificity compared to culture, and no cross-hybridization with other mycobacteria	5 pg of genomic DNA	5 min after application	[31]
	Sputum isolates (*n* = 45)	16 s rDNA for MTBC and MTB	Colorimetric by direct observation or spectrophotometry	100% concordance with culture	1 ng for PCR product and 40 ng for genomic DNA	1 h after DNA extraction	[32]
	Synthetic target ssDNA	rpoB gene of MTBC	Colorimetric	NA	NA	30 min	[33]
	Whole-blood samples	IS6110 locus for MTB	Colorimetric By RGB using smartphone application	NA	2.6 nM	1 h after DNA extraction	[34]
	Sputum isolates	rpoB locus for MTBC and MDRTB	Colorimetric by RGB analysis using a smartphone	100% concordance with culture	NA	Less than 50 min after DNA amplification	[35]
	Sputum, urine (*n* = 10), clinical isolates (*n* = 24)	Mutant alleles (*n* = 5) for MDRTB genes: katG315, inhA-15, rpoB526, embB306, rpsL43	SPR sensor to detect SPR angle changes	(92%–100%) sensitivity, (80%–100%) specificity compared to sequencing	5 × 10^–12^ M	Less than 5 h	[36]
	Sputum (*n* = 8) sample	S6110 locus for MTB	Electrochemical by conventional three-electrode system	100% concordance with PCR	1 × 10^–9^ M	NA	[37]
	Reference isolates	RpoB locus for MTB	Electrochemical by DPV	NA	1 CFU	Less than 90 min	[38]
	Sputum urine CSF (*n* = 24)	MTBC and MAC	SPR sensor to detect SPR angle changes	95.8% concordance with PCR	4.2 × 10^4^ CFU for MTB and 3.7 × 10^4^ CFU mL for *M*. *bovis*	NA	[39]
	*Mycobacterium* samples (*n* = 72)	Unamplified MTB16s rDNA	AuNP-based FRET system	Sensitivity and specificity of 98.6% and 90%, respectively, compared to PCR	3 ng/μL	15 min	[40]
	Synthetic target ssDNA	IS6110 locus for TB	Colorimetric	NA	1.95 × 10^−2^ ng/mL	60 min	[41]
	Synthetic target ssDNA	MTB	Cyclic voltammetry and chronoamperometric methods	92% specificity to MTB target DNA as compared with noncomplementary DNA	0.1 fM	NA	[42]
	Synthetic DNA	IS6110 locus for MTB	Electrical frequency changes by the proposed sensor	The current analytical system displays 12-fold higher detection of target DNA compared to the single mismatched DNA and other strands	10 fM	1 min	[43]
	Sputum specimens	IS6110 locus for MTB	Differential pulse voltammetry	88.6%	3 fM	NA	[44]
	Sputum samples (*n* = 60) and BACTEC MGIT (reference test)	IS6110 locus for MTB	TB nanogold assay	Sensitivity and specificity of 95% and 100%, respectively	11.2 ng/µL	2 h	[45]
	Sputum samples (*n* = 40) and reference strain	MTB	Electrical frequency changes by the proposed sensor	High affinity and high specificity	100 CFU/mL	2 h	[46]
	Ten bovine (from lymph nodes) and human (from phlegm) specimens infected by *Mycobacterium bovis* and MTB, respectively	IS6110 locus for MTB	Lateral flow test strips	No cross-reaction with other strains (*M. bovis* as negative samples)	NA	10 min after PCR reaction	[47]
	Clinical isolates	MTB	Colorimetric by direct observation or spectrophotometer	100% concordance with PCR-RFLP	10 CFU/mL of MTBC	Less than 1 h	[48]
	Synthetic target DNA	MTB	Dark-field microscopic imaging	NA	10 fM	NA	[49]
	Sputum specimens (*n* = 52)	IS6110 locus for MTB	Colorimetric probe-based biosensor	100% sensitivity and specificity for detection of total sputum DNA	4.8 × 10^−3^ ng/reaction	Less than 15 min	[50]

Magnetic	Synthetic target ssDNA	MTB	Electrochemical by DPV	NA	7.96 × 10^−13^ M	NA	[51]
	MTB-spiked sputum and real sputum samples	FadeE15 locus for MTB, rpoB locus for MDRTB	NMR measurement by the microfluidic device	100% concordance for real samples	10^4^ CFU/1 mL spikedsputum	Less than 2.5 h	[52]
	Clinical isolates	16-23S locus for MTBC rpoB locus for MDRTB	Frequency-dependent measurement by a portable AC susceptometer	100% concordance to culture results	10 amol	NA	[53]
	Clinical isolates	TB DNA	Colorimetric by direct observation or spectrophotometer	70%–120% of RT-PCR	6 ng µL^−1^	NA	[54]
	Sputum sample (*n* = 500)	Glycans and glycan-binding proteins	Nanoparticle-based colorimetric biosensing assay	95%–100%, 100%, and 100% sensitivity, specificity, and accuracy, respectively, for detection of MTB compared to SSM	10^2^ CFU/mL of MTB	10–20 min	[55]

Cadmium selenite quantum dots	• BAL (*n* = 60) • Fecal (*n* = 20) • LNs and ilium (FFPE) sections	• 23S rRNA locus for mycobacteria genus • IS6110, IS900 for MTB and MAP	Fluorescence	• For BAL: 88.33% concordance with culture, 90%, concordance with qPCR • For fecal samples: 85% concordance to qPCR • FFPE sections: 69.69% concordance to qPCR	12.5 ng	Less than 2 h	[56]

Cadmium telluride quantum dots	Clinical isolates (*n* = 50)	ESAT-6 region for MTBC and *M*. *bovis*	FRET signal by spectrophotometer	94.2% sensitivity, 86.6% specificity, compared to culture	10 fg	NA	[57]

Cadmium sulfide and lead sulfide quantum Dots	MTB-spiked serum samples	MTB and HIV	Anodic stripping voltammetry measurement by an electrochemical workstation	94%−102% spiked recovery range from	0.2 fM	NA	[58]

Cobalt and reduced graphene oxide	Human blood	Methyl nicotinate (MN) of MTB	Differential pulse voltammetry	98%–102%	0.0004 mM	90 min	[59]

Graphene oxide	Sputum samples (*n* = 54)	IS6110 locus for MTBC	A low-cost fluorimeter	0.925 kappa agreement with RQ-PCR	NA	NA	[60]

Silver	Target DNA-spiked solution	MTB	ECL signal detected by electrochemical impedance spectroscopy	NA	0.03 fmol	NA	[61]

Streptavidin	Sputum samples (*n* = 176)	IS6110 locus for MTBC vs. NTM	Fluorescence by fluorescence microscopy	88.3% sensitivity, 91.8% specificity, 93.8% PPI, 84.8% NPV compared to culture	10 bacteria	Less than 6 h	[62]

Upconversion	Sputum samples (*n* = 204)	IS6110 locus for MTBC	FTRET By a fiber-optic spectrometer	0.8464 Kappa agreement with PCR	10^2^ copies/μL	NA	[63]

HAPNPs/PPY/MWCNT nanocomposite	Spiked MTB sputum samples	IS6110 locus for MTB	Electrochemical by DPV	Excellent specificity for the detection of whole genome of MTB H37Rv	0.141 nM	NA	[64]

rGO-PDA-AuNP nanocomposite	Synthetic target ssDNA	MTB	Cyclic voltammetry and linear sweep voltammetry	2.12 × 10^−3^ mA µM^−1^ sensitivity	0.1 × 10^−7^ µM	5 s	[65]

*Note:* ECL, electrogenerated chemiluminescence (a) non-cross-linking approach and (b) cross-linking approach; RFLP, restriction fragment length polymorphism, dependent isothermal amplification.

Abbreviations: ADC, analog-to-digital converter; AuNP, gold nanoparticle; BAL, bronchoalveolar lavage; DPV, differential pulse voltammetry; dUTP, deoxyuridine triphosphate; EIS, electrochemical impedance spectroscopy; FFPE, formalin-fixed paraffin-embedded; FRET, fluorescence resonance energy transfer; GCE, glass carbon electrode; HAPNPs, hydroxyapatite nanoparticles; MAC, *Mycobacterium avium* complex; MAP, *Mycobacterium avium* subsp. *paratuberculosis*; MDRTB, multiple drug-resistant TB; MTB, *Mycobacterium tuberculosis*; MTBC, *mycobacterium tuberculosis* complex; MWCNTs, multiwalled carbon nanotubes; *n*, number of tested samples; NA, not available; NMR, nuclear magnetic resonance; PCB, printed circuit board for signal; PPy, polypyrrole; qPCR, real-time PCR; RQ-PCR, real-time quantitative PCR; SPR, surface plasmon resonance; ssDNA, single-strand DNA; SSM, sputum smear microscopy; SWV, square-wave voltammetric.

**Table 2:** Summarizes antigen-/protein-based detection.

Nanoparticle type	Sample type	Target	Signal detection method	Clinical performance	Lower detection limit	Turnaround time	Reference
Gold	Anti-LAM-spiked and HIgG-spiked solution	LAM antigen for MTB	Electrochemical by DPV measurements	No cross-reaction with human IgG	5.3 ng/mL	NA	[66]
	Whole blood and serum spiked with MTB 38 kDa monoclonal antibody	MTB 38 kDa monoclonal antibody for MTB	Cytometry-based immunochromatography	No cross-reaction with other analytes in the sample	5 ng/mL	NA	[67]
	IFN-γ-spiked solutions	IFN-γ for MTB	Electrochemical by DPV measurements	−4.37%–2.82% relative deviation to ELISA	0.048 pg/mL	NA	[68]
	Culture filtrates (at 96-h period)	CFP10-ESAT6 antigen and DNA sequence for MTB	Electrical frequency changes by the proposed sensor	No cross-reaction with NTM and other bacteria	10^3^ CFU/mL	96.3 h	[69]
	Synthetic antibodies	ESAT-6 protein in MTB	ELISA	~7.5-fold improved detection compared to the conventional ELISA	1 nM	NA	[70]
	MTB	16-kDa heat shock protein	Nanogapped impedimetric immunosensor	Higher sensitivity is demonstrated	100 fM	NA	[71]
	MTB	MPT64 antigen of MTB	Electrochemical by DPV and voltammetry measurements	The sensor has high specificity and reproducibility	10 fg·mL^−1^	NA	[72]
	Synthetic antibodies	ESAT-6 antigen of MTB	Colorimetric assay	NA	1.25 pM	NA	[73]
	Spiked saliva samples	ESAT-6 (Rv3875) of MTB	Electrical frequency changes by the proposed sensor	10^5^-fold higher sensitivity than ELISA	10 fg/mL	NA	[74]
	Sputum samples (PTB = 49 and EPTB = 42)	CFP-10 (Rv3874) of MTB	Electrical frequency changes by the proposed sensor	Sensitivities of 83.7% and 76.2% were observed in PTB and EPTB patients, respectively, with specificities of 93.5%–93.8% (*n* = 63) compared to RT-I-PCR	3 × 10^−2^ pg/mL	NA	[75]
	Synthetic antibodies	CFP-10 and ESAT-6 antigen	Lateral flow immunoassays	NA	0.0625 ng/mL for ESAT-6 and 7.69 ng/mL for CFP-10	15 min	[76]

Magnetic	BCG-spiked sputum	BCG	NMR measurements by NMR spectroscopy	100% concordance with culture	20 CFU	Less than 30 min	[77]
	BCG broth (preincubated with human THP-1 cell culture)	EPTB Pre-activated THP-1 monocytes for EPTB	NMR measurements by NMR spectroscopy	NA	NA	• In vitro assay: 1 h after adding SPIOTBsAB	[78]

Silica	Spiked sputum sample	MTB by protein A-anti-MTB AB affinity	Fluorescent nanoparticle-based indirect immunofluorescence microscopy (FNP-IIFM)	97.1% sensitivity, 95.5% specificity, 95.5% PPV, 96.5% NPV compared to culture	100 CFU/mL	Less than 4 h	[79]
	• Suspensions of reference isolates • MTB-spiked urine (*n* = 10)	MTB	Fluorescence by two-color flow cytometry	Better specificity than conventional flow cytometry	3.5 × 10^3^ cells/mL For isolates 3.0 × 10^4^ cells/mL for urines	Less than 2 h	[80]
	Putum samples (*n* = 153)	MTB by protein A-anti-MTB AB affinity	Fluorescence by fluorescence microscopy or confocal microscopy	97.1% sensitivity, 91.35% specificity	100 CFU/mL	Less than 4 h	[81]
Au@PB	Human serum	MPT64 antigen	Electrochemistry ELISA	97.4% to 110%	21 fg·mL^−1^	90 min	[82]

Au@Pt	Human serum	MPT64 antigen	Electrochemical by DPV	96% to 102%	0.33 fg/mL	NA	[83]

Au@Pd	Human serum (*n* = 40 samples) and urine (*n* = 28 samples)	CFP-10 antigens secreted from MTB	Nanozyme probe-based colorimetric ELISA	94%−106% in human serum; 93%−105% in urine	5.6 × 10^−12^ g mL^−1^	3 h	[84]

Au and Cu	Urine samples (*n* = 52) (21 with active TB, 16 with LTB, 6 with NTM lung disease, and 9 healthy without TB infection)	Ag85B and CFP10 antigens of MTB	Direct immunoblotting technique with copper and gold augmentation	The sensitivity for the detection of Ag85B and CFP10 was 90.5% and 76.2%, while the specificity was 86.7% and 66.7%, respectively	0.93 and 0.21 ng/mL by a phone camera and 1.56 and 0.75 ng/mL by the naked eye, respectively, are for Au and Cu	NA	[85]

Cadmium selenite quantum dots	Reference isolates	Mycobacteria	Fluorescence by direct observation or a spectrofluorometer	100% discrimination between mycobacteria and nonmycobacteria	10^3^ bacteria/mL (by spectrofluorometer • 10^4^ bacteria/mL by direct observation	NA	[86]

Detonation nanodiamond	MGIT isolates (*n* = 42)	CFP-10 for MTBC vs. NTM discrimination	MALDI-TOF MS	100% sensitivity, 98% specificity, compared to culture biochemical tests	0.09 μg/mL	1 h	[87]

Sensibeads and chemibeads	Blood (*n* = 72)	IFN-γ released by presensitized T cells for LTB	Chemiluminescence by a high-throughput homogeneous luminescence immunoassay instrument	97.2% concordance with T-SPOT	19.0 mIU/mL	30 min	[88]

Fe_3_O_4_@Ag/GQD nanocomposites	Spiked urine samples	CFP-10 antibody of MTB	Electrochemical by DPV measurements	CFP-10 recovery was between 93.76% and 106%, with RSD values ranging from 3.06% to 9.50%	0.33 ng/mL	NA	[89]

Abbreviations: Ab, antibody; Ag-Ab, antigen–antibody; BCG, Bacille Calmette–Guerin; DPV, differential pulse voltammetry; EPTB, extrapulmonary tuberculosis; GQD, graphene quantum dots; HIgG, human IgG; IDE, interdigital electrode; IFN-γ, interferon-γ; LAM, lipoarabinomannan; LTB, latent TB; MALDI-TOF MS, matrix-assisted laser desorption ionization time-of-flight mass spectrometry; MBs, magnetic beads; MRI, magnetic resonance imaging; MTB, *Mycobacterium tuberculosis*; MTBC, *Mycobacterium tuberculosis* complex; NA, not available; NMR, nuclear magnetic resonance; NTM, nontuberculous mycobacterium; PTB, pulmonary tuberculosis; RSD, relative standard deviation; TBsAB, TB surface antibody.

**Table 3:** Comparison of nanoparticle types for TB detection.

Feature/nanoparticle	Gold NPs	Magnetic NPs	Quantum dots
Detection mode	Colorimetric (plasmonic shift) [34, 90]	Magnetic separation + electrochemical sensing [91, 92]	Fluorescence (photoluminescence) [93]
Sensitivity	Moderate (~340 fmol to 195 ng/mL) [41, 90]	High (0.5 fg DNA or ~10^3^ CFU/mL) [91, 92]	Very high (~0.13 amol/L) [93]
Multiplexing capability	Low (one target per assay) [90]	Moderate (limited by readout channels) [92]	High (spectrally tunable QDs) [91]
Point-of-care potential	High (easy to interpret visually, minimal tools) [34, 90]	Moderate (requires magnets/electrodes) [91]	Low to moderate (requires fluorescence reader) [93]
Biocompatibility	High (inert, stable, nontoxic) [90]	Moderate (depends on coating/surface chemistry) [92]	Low (potential heavy metal toxicity) [94, 95]
Ease of use	High (simple protocols, rapid) [34, 90]	Moderate (requires handling with magnetic tools) [91]	Low (requires expertise in optics and instrumentation) [93]
Cost	Low (cheap synthesis, no instruments) [34, 90]	Moderate (costs tied to magnetic hardware and bioconjugates) [92]	High (QD synthesis, conjugation, detectors are expensive) [91, 92]
Instrument requirement	Minimal (naked-eye readout) [34, 90]	Requires magnet or electrochemical reader [91, 92]	Requires fluorescence microscopy or detectors [93, 94]

## Data Availability

Data sharing is not applicable to this article as no new data were created or analyzed in this study.
